# A retrospective cohort study of methylprednisolone therapy in severe patients with COVID-19 pneumonia

**DOI:** 10.1038/s41392-020-0158-2

**Published:** 2020-04-28

**Authors:** Yin Wang, Weiwei Jiang, Qi He, Cheng Wang, Baoju Wang, Pan Zhou, Nianguo Dong, Qiaoxia Tong

**Affiliations:** 1 Department of Cardiovascular Surgery, Union Hospital, Tongji Medical College, Huazhong University of Science and Technology, Wuhan, 43000 China; 2 Department of Gastroenterology, Union Hospital, Tongji Medical College, Huazhong University of Science and Technology, Wuhan, 43000 China; 3 Department of Rheumatology, Union Hospital, Tongji Medical College, Huazhong University of Science and Technology, Wuhan, 43000 China; 4 Department of Infectious Diseases, Union Hospital, Tongji Medical College, Huazhong University of Science and Technology, Wuhan, 43000 China; 5 Department of Hand Surgery, Union Hospital, Tongji Medical College, Huazhong University of Science and Technology, Wuhan, 43000 China

**Keywords:** Infectious diseases, Respiratory tract diseases

**Dear Editor,**

Corona Virus Disease 2019 (COVID-19) was first reported in late December 2019, in Wuhan, China. There are over 1,800,000 confirmed cases worldwide.^1^ The pathological process of severe COVID-19 pneumonia is an inflammation reaction characterized by the destruction of the deep airway and alveolar.^2^ It is currently considered that lung injury is not only associated with the direct virus-induced damage, but also the immune responses triggered by COVID-19 that lead to the activation of immune cells to release a large number of pro- and anti-inflammatory cytokines. Histologic examination has shown diffuse alveolar damage and mucinous exudate, which is similar to acute respiratory distress syndrome.^2^ Aggravation of symptoms always occurs during 5–7 days after onset in patients with COVID-19 pneumonia and severe cases develop rapidly to acute respiratory failure.^3^ Therefore, it is important to strengthen the treatment to suppress the pro-inflammatory response and control the cytokine storm at this stage. Methylprednisolone are the classical immunosuppressive drugs, which are important to stop or delay the progress of the pneumonia, and have been proved to be effective for the treatment of acute respiratory distress syndrome (ARDS).

In a recent study, Wu et al.^4^ found the administration of methylprednisolone appeared to reduce the risk of death in COVID-19 pneumonia patients with ARDS, however, of those who received methylprednisolone treatment, 23 of 50 patients died. This is a rather high mortality rate of ~50%; therefore, in terms of the indication, timing, dosage and duration, the application of methylprednisolone warrants further investigation. In another study, Zhou et al.^5^ endorsed the potential benefits of low-dose corticosteroids treatment in a subset of critically ill patients with COVID-19 pneumonia, however, the data was limited to only 15 patients and no control group. Although this is an important issue with regard to the challenges in the treatment of severe COVID-19 pneumonia, the clinical applicability of methylprednisolone needs to be tempered owing to the unanswered questions that remain. To address this issue, we performed a retrospective cohort study comparing the clinical outcomes of COVID-19 pneumonia patients with or without methylprednisolone treatment.

We studied 46 severe patients with COVID-19 pneumonia at the isolation ward of Union Hospital of Huazhong University of Science and Technology, Wuhan, China, from January 20 to February 25, 2020. The clinical classification is based on the coronavirus pneumonia diagnosis and treatment plan (trial version 5) developed by the National Health Committee of the People’s Republic of China. Severe case was defined when any of the following criteria was met: (1) respiratory distress, respiratory rate per min ≥ 30; (2) in the resting state, means oxygen saturation ≤ 93%; (3) arterial blood oxygen partial pressure/oxygen concentration ≤ 300 mmHg.

The age, sex, comorbidities, clinical, and laboratory parameters of these patients on admission are shown in Supplementary Table S1. Three (5.4%) patients died during the hospitalization, and the other 43 patients were successfully discharged. Oxygen therapy, antiviral therapy (a-interferon, Kaletra [lopinavir/ritonavir]), immunoenhancement therapy (thymosin), prevention of bacterial infection, relieving cough eliminating phlegm, and nutritional support were commonly used for all of the 46 patients; while, 26 of them received extra low-dose methylprednisolone treatment with the dosage of 1–2 mg/kg/day for 5–7 days via intravenous injection. The specific dosage and duration of methylprednisolone for the patients were determined according to the clinical manifestations, leukocyte count, lymphocyte count, inflammatory index, and lesion range. There was no significant difference of age, sex, comorbidities, clinical, or laboratory parameters between the two groups (Supplementary Data Table S1).

The median of SpO_2_ at rest on admission was similar between the two groups. All of the 46 patients received oxygen therapy. The dynamic change of SpO_2_ was shown in Fig. 1a, and the patients with methylprednisolone treatment had a faster improvement of SpO_2_. Moreover, patients without methylprednisolone treatment had significantly longer interval of using supplemental oxygen therapy than those with methylprednisolone treatment (8 days [interquartile range (IQR) 7–10] vs. 14 days (IQR 10–16); *P* < 0.001). Regarding the most intense level of oxygen support, the patients who received methylprednisolone treatment were less likely to be developed to receive the mechanical ventilation (*P* = 0.05); 35% (7/20) patients without methylprednisolone treatment developed to receive the mechanical ventilation (5 non-invasive ventilator [NIV], 2 IMV [invasive ventilator], no IMV with extracorporeal membrane oxygenation (ECMO)); while only 11.5% (3/26) patients with methylprednisolone treatment developed to receive the mechanical ventilation (2 NMV, 1 IMV, no IMV with ECMO).

**Fig. 1:**
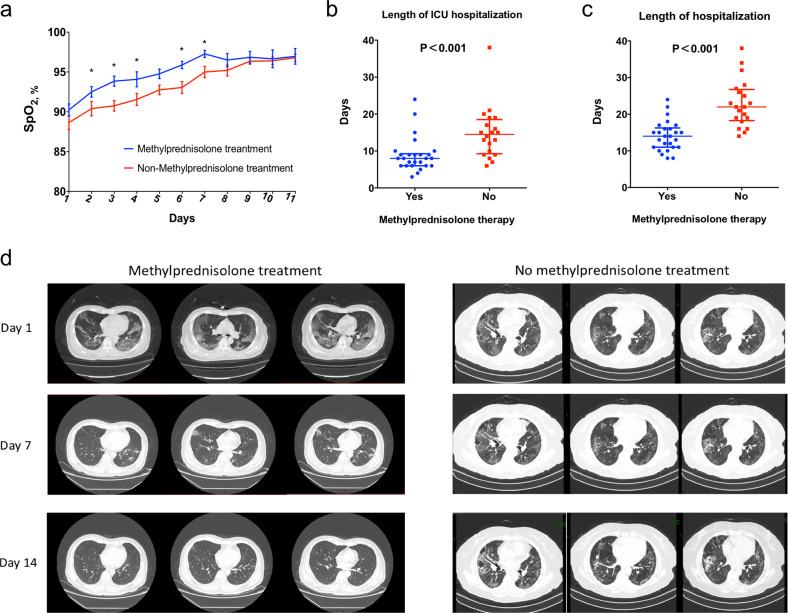
Comparison of the clinical outcomes between severe COVID-19 pneumonia patients with and without methylprednisolone treatment. **a** Dynamic change of SpO_2_ at rest; **b** length of ICU hospitalization; **c** length of hospitalization; **d** images of chest CT scan on day 1, 7, and 14 after hospitalization

The laboratory test was performed every 3 days and methylprednisolone treatment was associated with a faster decrease in C-reactive protein and interleukin-6 (Fig. 1c, d), while no significant difference was observed in other inflammatory indexes (Supplementary Fig. S1). Two deaths occurred in the patients with methylprednisolone treatment and one was in the patients without methylprednisolone treatment. No significant difference of mortality rate was observed between the two groups (*P* = 0.714). Additionally, the length of ICU hospitalization was significantly shorter in patients with methylprednisolone treatment (8 days [IQR 6–9] vs. 15 days [IQR 9–19]; *P* < 0.001, Fig. 1b) and length of hospitalization (14 days [IQR 11–16] vs. 22 days [IQR 18–26]; *P* < 0.001, Fig. 1c). In terms of chest CT scan on day 7 and 14, the absorption degree of the focus was significantly better in the patients with methylprednisolone treatment (Fig. 1d).

Methylprednisolone treatment-induced complications is another major concern. The most common complication caused by methylprednisolone is secondary infection. In our experience, once the secondary infection occurs in severe patients with COVID-19 pneumonia, sensitive and full-dose antibacterial drugs should be immediately added. Secondly, the use of immune regulators (human immunoglobulin) can enhance the patients’ immune function. In our experience, human immunoglobulin was usually used in the critical patients with a dosage of 10–20 g/day for 7–10 days as the pulse therapy. Thirdly, thymosin secreted by thymic epithelial cells can promote the maturation of T-lymphocytes and regulate function of cellular immunity. We suggest using thymosin during hospitalization, and the course of treatment can be determined according to the results of lymphocyte test. Benefiting from condition monitoring and refined management, no serious methylprednisolone treatment-induced complications were observed in the present cohort.

In conclusion, early, low-dose and short-term application of methylprednisolone was associated with better clinical outcomes in severe patients with COVID-19 pneumonia, and should be considered before the occurrence of ARDS. Nevertheless, future randomized controlled trials are desperately in need to confirm these findings and further study the mid- and long-term outcomes after discharge.

## Supplementary information

Supplementary table and figures

## Data Availability

The data used to support the findings of this study are included within the article and Supplementary Materials.
