# Supplementary material for: A retrospective cohort study of methylprednisolone therapy in severe patients with COVID-19 pneumonia
Source: Signal Transduct Target Ther. 2020 Apr 28;5:57. doi: 10.1038/s41392-020-0158-2 (PMC7186116; doi:10.1038/s41392-020-0158-2)
Supplement: Supplementary file 1 — Supplementary table and figures [file 41392_2020_158_MOESM1_ESM.docx]

Supplementary Materials for

**Application of methylprednisolone in severe patients with COVID-19 pneumonia: a retrospective cohort study**

**Yin Wang^1^, Weiwei Jiang^3^, Qi He^3^, Cheng Wang^4^, Baoju Liu^2^, Pan Zhou^5^, Nianguo Dong^1^**^†^**, Qiaoxia Tong^2^**^†^

^1^Department of Cardiovascular Surgery, Union Hospital, Tongji Medical College, Huazhong University of Science and Technology, Wuhan, 43000, China

^2^Department of Gastroenterology, Union Hospital, Tongji Medical College, Huazhong University of Science and Technology, Wuhan, 43000, China

^3^Department of Rheumatology, Union Hospital, Tongji Medical College, Huazhong University of Science and Technology, Wuhan, 43000, China

^4^Department of Infectious Diseases, Union Hospital, Tongji Medical College, Huazhong University of Science and Technology, Wuhan, 43000, China

^5^Department of Hand Surgery, Union Hospital, Tongji Medical College, Huazhong University of Science and Technology, Wuhan, 43000, China

†Correspondence authors

**Running Title: Methylprednisolone treatment in COVID-19 pneumonia**

**Corresponding Author**:

Qiaoxia Tong, M.D, Ph.D.

Address: Jiefang Avenue 1277#, Wuhan, Hubei, 430000, China

Institution: Department of Infectious Diseases, Union Hospital, Tongji Medical College, Huazhong University of Science and Technology, China

Phone number: 008613971311983

Fax number: 02785351609

E-mail: [2013xh0859@hust.edu.cn](mailto:2013xh0859@hust.edu.cn)

Nianguo Dong, M.D, Ph.D.

Address: Jiefang Avenue 1277#, Wuhan, Hubei, 430000, China

Institution: Department of Cardiovascular Surgery, Union Hospital, Tongji Medical College, Huazhong University of Science and Technology, China

Phone number: 008613971181551

Fax number: 02785351613

E-mail: dongnianguo63@gmail.com

Table 1 Clinical characters and main laboratory parameters of 46 patients with severe COVID-19 pneumonia

|  | All patients | Methylprednisolone treatment | |  |
| --- | --- | --- | --- | --- |
|  | (n=46) | Yes(n=26) | No(n=20) | P value |
| Age, years | 54(48,64) | 54(48,63) | 53(48,63) | 0.916 |
| Male | 26(57) | 16(62) | 10(50) | 0.434 |
| Chronic cardiac disease, n(%) | 6(13) | 3(12) | 3(15) | 0.729 |
| Chronic pulmonary disease, n(%) | 3(6.5) | 1(3.9) | 2(10) | 0.402 |
| Cerebrovascular disease, n(%) | 2(4.3) | 0(0) | 2(10) | 0.099 |
| Malignancy, n(%) | 2(4.3) | 1(3.9) | 1(5) | 0.849 |
| Diabetes | 4(8.7) | 3(12) | 1(5) | 0.435 |
| Hypertension, n(%) | 14(30) | 8(31) | 6(30) | 0.955 |
| Temperature, ℃ | 37.6(36.8,38.4) | 37.6(36.7,38.0) | 38.2(36.8,38.6) | 0.221 |
| Heart rate, per min | 86(74,96) | 86(69,97) | 86(82,96) | 0.598 |
| SBP, mmHg | 132(121,141) | 135(121,140) | 128(120,144) | 0.584 |
| DBP, mmHg | 77(70,85) | 77(70,85) | 77(70,86) | 0.438 |
| Respiratory frequency, per min | 26(21,31) | 28(21,36) | 24(20,30) | 0.039 |
| SpO_2_ at rest, % | 91(86,92) | 91(88,92) | 90(85,92) | 0.206 |
| WBC, G/L | 7.74(5.24,9.96) | 7.47(4.91,10.31) | 7.83(5.44,9.65) | 0.979 |
| PMN, G/L | 5.53(3.85,7.87) | 5.59(3.59,8.27) | 5.35(3.85,7.87) | 0.823 |
| LYM, G/L | 0.86(0.58,1.15) | 0.77(0.50,1.14) | 0.91(0.68,1.22) | 0.176 |
| CPR, mg/L | 61.5(14.4,109.9) | 78.9(33.7,115.6) | 61.3(15.7,109.9) | 0.646 |
| PCT, mg/L | 0.55(0.14,1.09) | 0.60(0.15,1.09) | 0.29(0.13,1.05) | 0.495 |
| IL-2, pg/ml | 2.77(2.32,3.55) | 2.89(2.45,3.54) | 2.97(2.45,3.59) | 0.731 |
| IL-4, pg/ml | 2.17(1.87,2.61) | 2.45(2.05,2.70) | 2.14(1.89,2.46) | 0.213 |
| IL-6, pg/ml | 18.4(10.4,53.0) | 18.9(13.7,60.1) | 23.7(15.3,56.4) | 0.941 |
| IL-10, pg/ml | 6.7(3.9,8.1) | 6.9(5.8,9.0) | 6.7(3.9,8.1) | 0.123 |
| FER, µg/L | 787(300,1057) | 528(300,1253) | 793(306,985) | 0.976 |

Data are n (%), or median (interquartile range). 2019-nCoV, 2019 novel coronavirus; SBP, Systolic Pressure; DBP, diastolic pressure; SpO2, oxygen saturation; WBC, white blood cell PMN, neutrophil; LYM, lymphocyte; CRP, C-reactive protein, PCT, procalcitonin; IL-2, interleukin-2; IL-4, interleukin-4; IL-6, interleukin-6; IL-10, interleukin-10; FER, ferroprotein


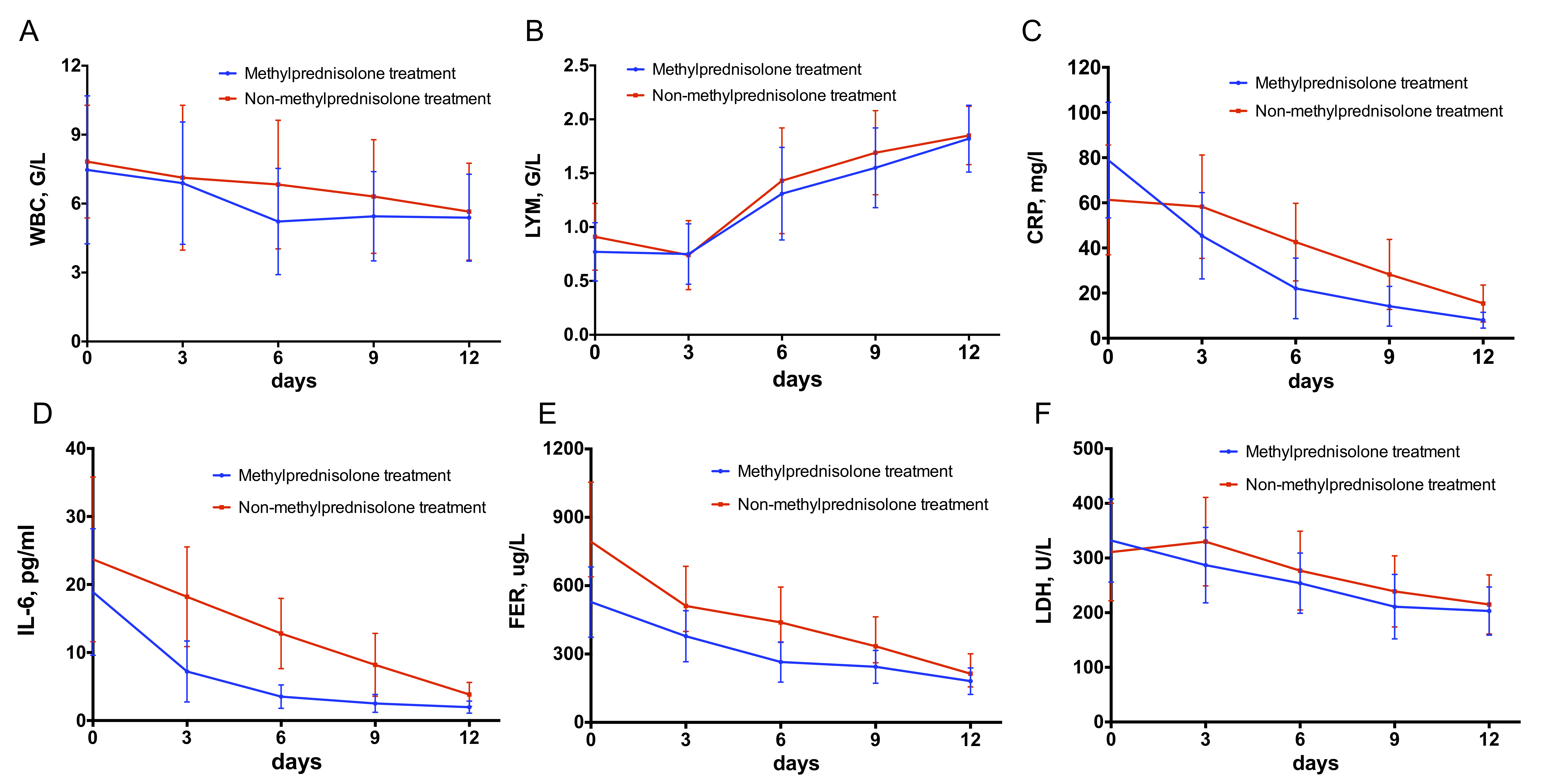


Figure S1 Comparison of the dynamic change of main inflammatory indexes between severe COVID-19 pneumonia patients with and without methylprednisolone treatment.(A) WBC; (B)LYM; (C)CRP; (D)IL-6; (E)FER; (F)LDH
